# The clinical efficacy of azathioprine as maintenance treatment for autoimmune pancreatitis: a systematic review and meta-analysis

**DOI:** 10.1007/s00535-021-01817-9

**Published:** 2021-08-24

**Authors:** Yoshiharu Masaki, Hiroshi Nakase, Yoshihisa Tsuji, Masanori Nojima, Kyoko Shimizu, Nobumasa Mizuno, Tsukasa Ikeura, Kazushige Uchida, Akio Ido, Yuzo Kodama, Hiroshi Seno, Kazuichi Okazaki, Seiji Nakamura, Atsushi Masamune

**Affiliations:** 1 Department of Gastroenterology and Hepatology, Sapporo Medical University School of Medicine, S-1, W-16, Chuo-ku, Sapporo, Hokkaido 060-8543 Japan; 2 Department of General Medicine, Sapporo Medical University School of Medicine, Sapporo, Japan; 3 Center for Translational Research, The Institute of Medical Science, The University of Tokyo, Tokyo, Japan; 4 Institute of Gastroenterology, Tokyo Women’s Medical University, Tokyo, Japan; 5 Department of Gastroenterology, Aichi Cancer Center Hospital, Nagoya, Japan; 6 The Third Department of Internal Medicine, Kansai Medical University, Hirakata, Japan; 7 Department of Gastroenterology and Hepatology, Kochi Medical School, Kochi University, Nankoku, Japan; 8 Digestive and Lifestyle Diseases, Kagoshima University Graduate School of Medical and Dental Sciences, Kagoshima, Japan; 9 Department of Gastroenterology, Kobe University, Kobe, Japan; 10 Department of Gastroenterology and Hepatology, Kyoto University Graduate School of Medicine, Kyoto, Japan; 11 Kansai Medical University Kori Hospital, Neyagawa, Japan; 12 Section of Oral and Maxillofacial Oncology, Division of Maxillofacial Diagnostic and Surgical Sciences, Faculty of Dental Science, Kyushu University, Fukuoka, Japan; 13 Division of Gastroenterology, Tohoku University Graduate School of Medicine, Sendai, Japan

**Keywords:** AZA, Autoimmune pancreatitis, Steroid, Relapse, Meta-analysis

## Abstract

The effectiveness of azathioprine (AZA) in preventing relapse and maintaining autoimmune pancreatitis (AIP) remission has been reported; however, most of these studies are case series with no randomized control trials available in the literature. Therefore, this study performed a systematic review and meta-analysis of the existing literature on this subject to determine the clinical efficacy of AZA as maintenance therapy for AIP patients. A systematic search was performed to identify studies on the clinical efficacy of AZA as maintenance therapy in AIP patients. The crude multiple relapse rate was estimated to assess the ability of AZA to control relapses in AIP. Pooled estimates were obtained using a random-effects model with the DerSimonian-Laird method. We identified AIP patients who did not respond to initial steroid treatment, experienced steroid weaning failure, or those who relapsed during remission as refractory cases. After reviewing the studies, ten articles fulfilled the inclusion criteria and were selected for meta-analysis. Of all 4504 patients, 3534 patients were treated with steroids, and 346 patients were treated with AZA for relapsed AIP. In this meta-analysis, 14/73 (19.2%) patients receiving AZA for refractory AIP relapsed. Meanwhile, 14/47 (29.8%) patients without AZA experienced relapse. The integrated odds ratio for relapse risk in patients receiving AZA was estimated to be 0.52 (*p* = 0.15). This systematic review and meta-analysis demonstrated the efficacy of AZA in preventing relapse of AIP, which supports the use of AZA as a maintenance treatment in patients with AIP who relapse upon withdrawal of steroid therapy.

**Supplementary Information:**

The online version contains supplementary material available at 10.1007/s00535-021-01817-9.

## Introduction

With progress in research on autoimmune pancreatitis (AIP), the number of AIP patients in Japan has increased [1]. A total of 33% or more of AIP patients exhibit features of acute pancreatitis (AP) or chronic pancreatitis (CP) at presentation [2]. AIP has distinct clinical, serological, radiological, and histological features from other types of AP or CP, such as hereditary, alcoholic, and preduodenal pancreatitis. The pathogenesis of AIP is considered to involve autoimmunity based on the rapid resolution of signs and symptoms related to pancreatic inflammation after the initiation of corticosteroid (CS) treatment. However, the exact mechanism of AIP remains unclear.

Oral CS administration has been established as a first-line treatment for AIP. Unfortunately, 15–60% of patients experience disease relapse during the initial tapering of steroids and after steroid treatment, despite the high initial response rate. Regarding a clinical study on the prevention of AIP relapse, Kamisawa et al. examined the efficacy of low-dose daily prednisone (PSL) (2.5–10 mg) in AIP patients and reported that the relapse rates in the steroid continued group and the discontinued group were 23% and 34%, respectively (*p* = 0.048) [3]. Additionally, Masamune et al. conducted a randomized controlled trial to clarify the efficacy of maintenance CS therapy in patients with AIP [4]. This study demonstrated that maintenance therapy with CS at 5–7.5 mg/day for three years might decrease relapses in AIP patients compared to those who discontinued the treatment at 26 weeks. Based on these findings, the continuation of low-dose CS may be useful for maintaining AIP remission.

A large Japanese cohort study [5] of AIP patients reported that 1223 (84.4%) of 1449 patients with AIP received initial CS treatment, and maintenance steroid therapy in AIP was used in 85.0% of patients, with a mean duration (SD) of 38.8 months (31.5 months). On the other hand, immunomodulators (IMs) [azathioprine (AZA) or 6-MP] were administered to 59 of 1234 patients (4.8%). These data indicate that IMs are rarely used for steroid tapering in AIP patients, and long-term CS administration is favored. Kaplan–Meier analysis revealed that the AIP relapse rate was 14% after 3 years, 25% after 5 years, 40% after 10 years, and 50% after 15 years. Concerning the daily dose (mg) of PSL at the time of relapse, approximately half of patients relapsed under the CS-free condition, and 32.6% of patients relapsed despite being on more than 5 mg/day of PSL. Based on these results, it is essential to establish some type of remission maintenance therapy for early withdrawal of CS and prevention of relapse in AIP patients who are generally more commonly elderly. With respect to the COVID-19 pandemic, it has been suggested that elderly patients and long-term steroid use are associated with COVID-19 severity. Additionally, given the complications associated with the long-term use of CS, it is essential to maintain AIP patients' steroid-free remission. The efficacy of AZA in preventing relapse and maintaining remission of AIP has been reported. However, most of these publications are case series, and there have been no randomized controlled trials (RCTs). Therefore, this study performed a systematic review and meta-analysis of the existing literature on this subject to determine the clinical efficacy of AZA as maintenance therapy in AIP patients.

## Methods

### Study selection

This meta-analysis was performed following the Preferred Reporting Items for Systematic Reviews and Meta-Analyses (PRISMA) statement. Initially, a systematic search of the MEDLINE, EMBASE, and SCOPUS databases was performed using the following terms: “autoimmune pancreatitis,” “relapse”, “steroid therapy”, and “maintenance”. We screened abstracts presented at the primary relevant pancreatic conference proceedings (Digestive Disease Week, and American/European Pancreatic Club) over the past 5 years. The search included reports published from June 2004 through December 2020. Additionally, a manual investigation of all review article reference lists and primary studies was performed. We selected only the most recent and complete data when the results of a single study were reported in more than one publication.

Second, letters, commentaries, or unreliable references were omitted by reviewing titles and abstracts (Tables 1A and B). Then, if patient cohorts included in this study overlapped (in this case, only the more recent study was included) or if the follow-up time was shorter than six months, studies were also excluded.

**Table 1:** Study- and patient-level variables

(A) Study-level variables	
Last name of the first author	
Year of publication	
Region where the study was conducted	One country or international
Study design	Prospective or retrospective
Number of centers	Single or multiple
Diagnostic criteria	2002/updated JPS criteria, HiSORT, Asian, ICDC, etc
Number of enrolled patients (treatment with steroids)	
Definition of relapse	clinical and radiologic, radiologic, or undefined
Length of follow-up evaluation	

(B) Patient-level variables	
Age	
Sex	
Number of patients with AZA	
First line therapy for relapsed AIP	Re-steroid, AZA/re-steroids with AZA, Rituximab with/without AZA, other
Number of patients administered AZA for relapsed AIP	
Number of patients with treatment failure for relapsed AIP	

Third, studies were included in the meta-analysis if they met the following criteria: (1) patients with a record of diagnostic instruction (e.g., International Consensus Diagnostic Criteria [6], Mayo Clinic’s HiSORT criteria [7], Japanese Pancreas Society guidelines [8], or Asian diagnostic criteria [9], etc.) and (2) AZA was administered to some of the patients with relapsed AIP.

The primary purpose of this meta-analysis is to examine whether AZA can prevent more than two relapses. To do this, each study was scored, and the quality was assessed according to a score sheet. In general, most researchers define a study with the Newcastle–Ottawa Quality Assessment Scale [10] (NOS) scores ≥ 6/9 as a high quality. Based on this scale, studies that scored 12 (60% of full score) or greater were classified as high-quality, for this outcome, and those with scores lower than nine were classified as low-quality. We set cut-off value as scores of 60% or more (≥ 12/20) to guarantee the quality.

## Review of the literature

Study-level variables included the year of publication, the region where the study was conducted, study design, number of centers, diagnostic criteria, number of enrolled patients and treatment with steroids, the definition of relapse, and length of follow-up evaluation (Table 1A). Referring to a previous study, we classified definitions of relapse into 3 categories: undefined (studies in which a clear definition of relapse was not reported), radiologic, or both clinical and radiologic. Patient-level variables included age, sex, number of patients with AZA, first-line therapy for relapsed AIP, number of patients administered AZA for relapsed AIP, and number of patients with AZA failure for relapsed AIP (Table 1B). Additionally, we investigated the number of type1 AIP in patients treated with AZA. All of the enrolled studies were evaluated and classified by two independent investigators (Y.T. and Y.M.). We performed a systematic review assessing the efficacy of AZA to control relapses in AIP. Discrepancies among reviewers were not frequent (interobserver variation, < 10%) and were resolved by discussion.

### Study quality

Referring to a checklist based on a modified version of the NOS [10] and a previous study [11], studies were graded using the following parameters: (1) representative cohort, (2) ascertainment of exposure, (3) demonstration that outcome of interest was not present, (4) initial steroid dose (daily), (5) dose of AZA (daily), (6) record of how to use immunosuppressor drugs, (7) assessment of the efficacy of treatments for relapsed AIP, (8) relapse definition, (9) sufficient follow-up evaluation, and (10) adequacy of follow-up schedule. Each parameter was assigned a numeric score from 0 to 2 (Table 2).

**Table 2:** Criteria for study quality

Representative cohort	Items	Consecutively enrolled	Not consecutive/prospective	Not consecutive, retro, case / NA
	Risk of bias	Very low	Low	High
	Point	2	1	0
Ascertainment of exposure	Items	International criteria	National diagnostic criteria	Non-validated criteria/NA
	Risk of bias	Very low	Low	High
	Point	2	1	0
Demonstration that outcome of interest was not present	Items	Yes	No	NA
	Risk of bias	Very low	Low	High
	Point	2	1	0
Initial steroid dose (daily)	Items	0.6 mg/kg or 3–40 mg	1 mg/kg or more	NA
	Risk of bias	Very low	Low	High
	Point	2	1	0
Dose of AZA (daily)	Items	2–2.5 mg/kg or 50–100 mg	Less than 2 mg/kg or 50 mg	NA
	Risk of bias	Very low	Low	High
	Point	2	1	0
Record of how to use immunosupressor drugs	Items	Yes	No	NA
	Risk of bias	Very low	Low	High
	Point	2	1	0
Assessment of effectiveness of treatments for relapse AIP	Items	Yes	No/NA	
	Risk of bias	Very low	High	
	Point	2	0	
Relapse definition	Items	Clinical and radiologic relapse	Clinical or radiologic relapse	Not a clear
	Risk of bias	Very low	Low	High
	Point	2	1	0
Sufficient follow-up evaluation	Items	> 2 y	≤ 2 y	Undefined
	Risk of bias	Very low	Low	High
	Point	2	1	0
Adequacy of F/U schedule	Items	Definite schedule	Undefinite	
	Risk of bias	Very low	High	
	Point	2	0	

We assessed representative cohort, obeying recommendations of NOS, Cochran risk of bias domains, and previous paper regarding bias risk [12]. According to these recommendations, selection bias (prospective selection or not) is essential information to maintain the quality of study. A retrospective cohort study has a higher risk of researcher bias (e.g., confirmatory bias, question-order bias, and leading questions or wording bias). In this regard, we evaluated the representative cohort in accordance with the criteria mentioned in the most recent meta-analysis regarding AIP treatment [11].

Regarding the initial dose of steroid, according to an international consensus on the treatment of AIP, the 0.6 mg/kg and 1 mg/kg of PSL treatments may be the same scorers. On the other hand, in the majority of all the studies included in this meta-analysis, AIP patients had received PSL treatment with an initial dose of 30–40 mg/day, but not 1 mg/kg of PSL. In this meta-analysis with the limited number of observational cohort studies, we considered it necessary to reduce the bias associated with the heterogeneity of treatment as much as possible. In this regard, we defined a dosage of 30–40 mg/day or 0.6 mg/kg of steroid as point 2, and 1 mg/kg as point 1.

### Statistical analysis

Generally, AZA was not used in AIP patients who were naïve to CS therapy and was primarily used in patients who exhibited steroid unresponsiveness, steroid weaning failure or relapse during remission (refractory cases). Therefore, we compared the multiple relapse rate (2 or more relapses) in refractory patients receiving AZA plus re-initiation of steroid therapy to those receiving re-initiation of steroid therapy alone in this meta-analysis (Suppl. Figure 1). The crude multiple relapse rate was estimated to assess the efficacy of AZA for controlling relapses in AIP. Pooled estimates were obtained using a random-effects model. Heterogeneity was assessed using the Pearson chi-square test and the *I*^2^ statistic.

For all other analyses, a *p* value less than 0.05 was considered statistically significant. The amount of heterogeneity in the outcome explained by risk factors was evaluated using the *R*^2^ index.

Egger’s regression test was performed to evaluate the asymmetry of Begg’s funnel plot and potential publication bias.

All statistical analyses were performed using EZR (Saitama Medical Center, Jichi Medical University, Saitama, Japan), i.e., a graphical user interface for R (The R Foundation for Statistical Computing, Vienna, Austria), including the package “meta” for meta-analysis. More precisely, it is a modified version of the R commander designed to add statistical functions frequently used in biostatistics. For studies with a zero cell count, 0.5 was added to all cell frequencies in these studies.

## Results

### Literature search

**Fig. 1:**
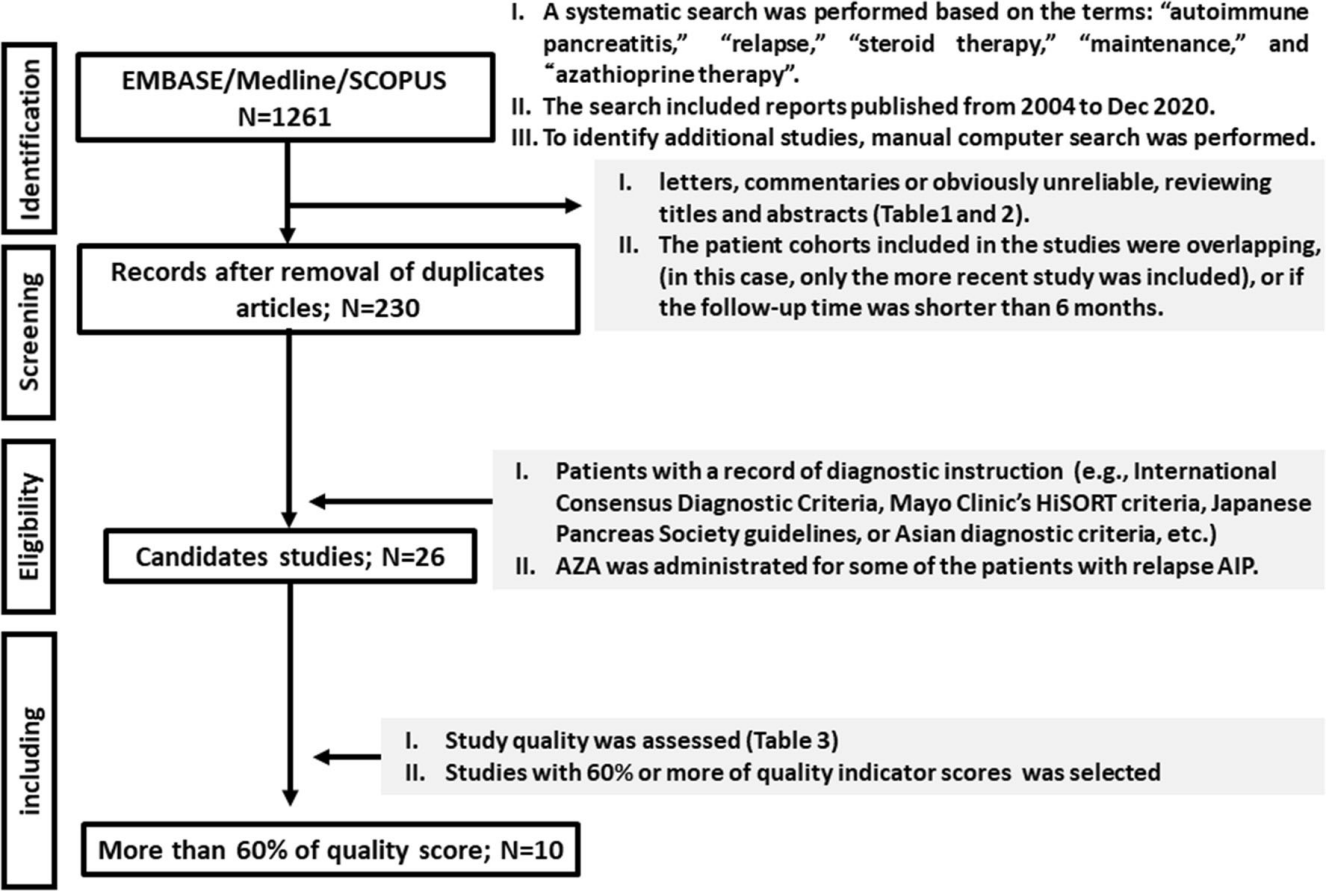
Study flow chart

Our primary search identified 1261 titles. After the removal of duplicate articles, 230 studies remained. Among these, 204 articles were excluded due to inconsistent aims in this study. Then, the remaining 26 studies [5, 13–37] were included in a qualitative synthesis, and the full-text was reviewed to establish eligibility for quantitative analysis (Tables 3, 4). After reviewing the studies, 10 full-text articles [13–22] fulfilled the inclusion criteria and were selected for meta-analysis (Fig. 1).

**Table 3:** Study- and patient-level characteristics for studies included in the meta-analysis

Author	Region	Number of centers	Design	Diagnostic criteria	Patients (with steroid)	Initial steroid dose (daily)	Patients with AZA (for Type1)	Dose of AZA (daily)	Definition of relapse	follow-up (Average month)
Huggett 2014	UK	Multi	Pro	ICDC	115 (98)	3–40 mg	41 (41)	2 mg/kg	R	32.5
Maire 2010	France	Single	Pro	HiSORT	44 (26)	40 mg	4 (2)	2.5 mg/kg	C and R	41
Pretis 2017	Italy	Single	Retro	ICDC	120 (114)	1 mg/kg	23 (20)	2–2.5 mg/kg	C and R	58.8( +), 32.4(–)*
Sandanayake 2009	UK	Single	Pro	International	28 (28)	30 mg/day	10 (–)	2 mg/kg	C and R	29
Soliman 2019	France	Single	Retro	Institutional	92 (71)	40 mg	19 (19)	2–2.5 mg/kg	C and R	33.6
Xin 2018	China	Single	Pro	International	183(101)	3–40 mg	4 (4)	50-100 mg	C and R	40
Buijs 2015	Holand	Multi	Pro	ICDC	107 (89)	3–40 mg	28 (–)	NA	C and R	74
Raina 2009	US	Single	Retro	HiSORT	26 (19)	40 mg	13 (–)	NA	Undefined	6
Rana 2018	India	Single	Retro	ICDC	18 (12)	40 mg	2 (1)	NA	C and R	8.5
Ikeura 2013	Italy	Single	Pro	ICDC	92 (74)	1 mg/kg	22 (–)	NA	C and R	> 24
Lee 2018	KOR	Single	Pro	ICDC	244 (138)	3–40 mg	NA	100 mg/day	R	60
Naitoh 2009	Japan	Single	Case	JPS 2002	1 (1)	30 mg	1 (–)	50 mg	C and R	at least 60
Kubota 2017	Japan	Multi	Retro	JPS 2002	510 (510)	30 mg	6 (6)	NA	C and R	61.1
Church 2007	UK	Single	Pro	ICDC	17 (9)	NA	4 (4)	1–2 mg/kg	R	51
Chatterjee 2014	UK	Single	Pro	HiSORT	22 (19)	NA	5 (–)	NA	Undefined	NA
Barresi 2020	Italy	Multi	Retro	ICDC	173 (149)	NA	19 (–)	NA	Undefined	NA
Hart 2016	US	Single	Pro	ICDC	43 (20)	Typically 40 mg	1 (–)	NA	C and R	34.8
Czakó 2011	Hungary	Multi	Retro	HiSORT	17 (15)	30–40 mg	1 (–)	1–2 mg/kg	Undefined	NA
Masamune 2020	Japan	Multi	Retro	JPS2011	1474 (1223)	0.6 mg/kg or 3–40 mg	47 (–)	NA	Undefined	NA
Lopez 2016	Spain	Multi	Retro	ICDC	52 (42)	NA	19 (19)	NA	C and R	45
Hart 2013	International	Multi	Retro	Each country	1064 (736)	0.6 mg/kg or 3–40 mg	68 (68)	NA	Undefined	> 24
El Euch 2017	Tunisia	Single	Case	NA	1 (1)	0.6 mg/kg	1 (1)	50 mg	Undefined	NA
Alidjan 2015	Netherlands	Single	Case	HiSORT	1 (1)	NA	1 (1)	50 mg	Undefined	12–36
Cousin 2018	France	Single	Case	NA	1 (1)	1 mg/kg	1 (0)	2 mg/kg	Undefined	NA
Lee 2019	UK	Single	Retro	NA	6 (4)	NA or 1 mg/kg	2 (1)	NA	Undefined	NA
Rasch 2015	Germany	Single	Retro	ICD-10	53 (33)	NA	4 (–)	NA	Undefined	NA

*Pro*; Prospective study, Retro; Retrospective study, Case; Case report, C; Clinical, R; Radiologic

*AZA ( +) and (–)

**Table 4:** Assessment of study quality

	Representative cohort	Ascertainment of exposure	Outcome of interest was not present	Initial steroid dose (daily)	Dose of AZA (daily)	Record of how to use immunosuppressor drugs	Assessment of effectiveness of treatments for relapse AIP	Relapse definition	Sufficient follow-up evaluation	Adequacy of F/U schedule	Quality score (Full = 20)
Huggett 2014	1	2	2	2	2	2	2	2	2	2	19
Maire 2010	2	1	2	2	2	1	2	2	2	2	18
Pretis 2017	2	2	2	1	2	2	0	2	2	2	17
Sandanayake 2009	1	2	2	2	2	2	0	2	2	2	17
Soliman 2019	2	2	2	2	2	2	0	2	0	2	16
Xin 2018	1	2	2	2	2	0	0	2	2	2	15
Buijs 2015	1	2	2	2	0	1	2	2	2	0	14
Raina 2009	2	1	2	2	0	2	2	2	0	0	13
Rana 2018	0	2	2	2	0	2	2	2	0	0	12
Ikeura 2013	2	2	2	1	0	1	0	2	2	0	12
Lee 2018	2	2	2	2	0	1	0	2	0	0	11
Naitoh 2009	0	1	2	2	2	0	0	2	2	0	11
Kubota 2017	0	1	2	2	0	1	0	2	2	0	10
Church 2007	0	2	2	2	1	1	0	2	0	0	10
Chatterjee 2014	2	1	2	2	0	1	0	0	2	0	10
Barresi 2020	0	2	2	1	0	2	0	0	2	0	9
Hart 2016	1	2	2	1	0	0	0	2	0	0	8
Czakó 2011	0	1	2	2	1	1	0	0	0	0	7
Masamune 2020	0	1	2	2	0	0	0	0	2	0	7
Lopez 2016	0	2	2	1	0	0	0	2	0	0	7
Hart 2013	0	2	2	2	0	0	0	0	0	0	6
El Euch 2017	0	0	2	2	2	0	0	0	0	0	6
Alidjan 2015	0	1	2	1	2	0	0	0	0	0	6
Cousin 2018	0	0	2	1	2	0	0	0	0	0	5
Lee 2019	0	0	2	1	0	0	0	0	0	0	3
Rasch 2015	0	0	2	0	0	0	0	0	0	0	2

### Study characteristics

The features of the studies selected are shown in Table 3. A total of 4504 AIP/IgG4-RD patients were included. Six studies [5, 18, 21, 23–25] were performed in Asian countries, and 20 [13–17, 19, 20, 22, 26–37] were conducted in Western countries. A single study [32] was multinational. Eighteen [14–18, 20–24, 26, 27, 29, 33–37] and eight [5, 13, 19, 25, 28, 30–32] studies were conducted as single- and multicenter endeavors, respectively.

Among all 26 studies, 10 [13, 14, 16, 18, 19, 22, 23, 26, 27, 29], 12 [5, 15, 17, 20, 21, 25, 28, 30–32, 36, 37], and 4 studies [24, 33–35] were prospective, retrospective, and case reports, respectively. Twenty-one studies [5, 13–31, 34] used diagnostic instructions (e.g., International Consensus Diagnostic Criteria, Mayo Clinic’s HiSORT criteria, Japanese Pancreas Society guidelines, or Asian diagnostic criteria).

Of all 4504 patients, 3534 patients were treated with steroids, and 346 patients were treated with AZA for relapsed AIP. Of the 346, at least 187 were type-1 AIP, while in the remaining patients, clinical information about the type of AIP was not clear. The number of patients treated with AZA varied greatly, ranging from 1 to 68 per study. Except for 6 cases without records, the initial daily doses of steroids in 16 and 4 studies were 0.6 mg/kg, 3–40 mg and 1 mg/kg, respectively. Among 13 studies with a record of the initial dose of AZA, the numbers of studies with doses for maintenance of 2.0–2.5 mg/kg or 50–100 mg and less than 2.0 mg/kg were 11 and 2, respectively. In three studies, relapse/rerelapse was radiologically defined, whereas, in 12 studies, it was both clinically and radiologically defined. In the remaining 11 studies, relapse was not clearly defined. In 15 studies, the follow-up evaluation term was longer than 2 years (in 8 studies, it was not specified). The details of patient numbers used in this analysis are shown in Supplementary Figs. 2–10 and Supplementary Tables 1–4.

### Meta-analysis of AZA to control relapse of AIP

The results of the quality assessment of the included studies are shown in Table 4. To maintain study quality, we selected ten studies with scores of 60% or more (≥ 12). Two studies in which the weight of the effect sizes was 0% [19, 20] and two studies in which the accurate number of patients receiving steroid therapy for two or more relapses was unclear [18, 22] were excluded from this analysis.

**Fig. 2:**
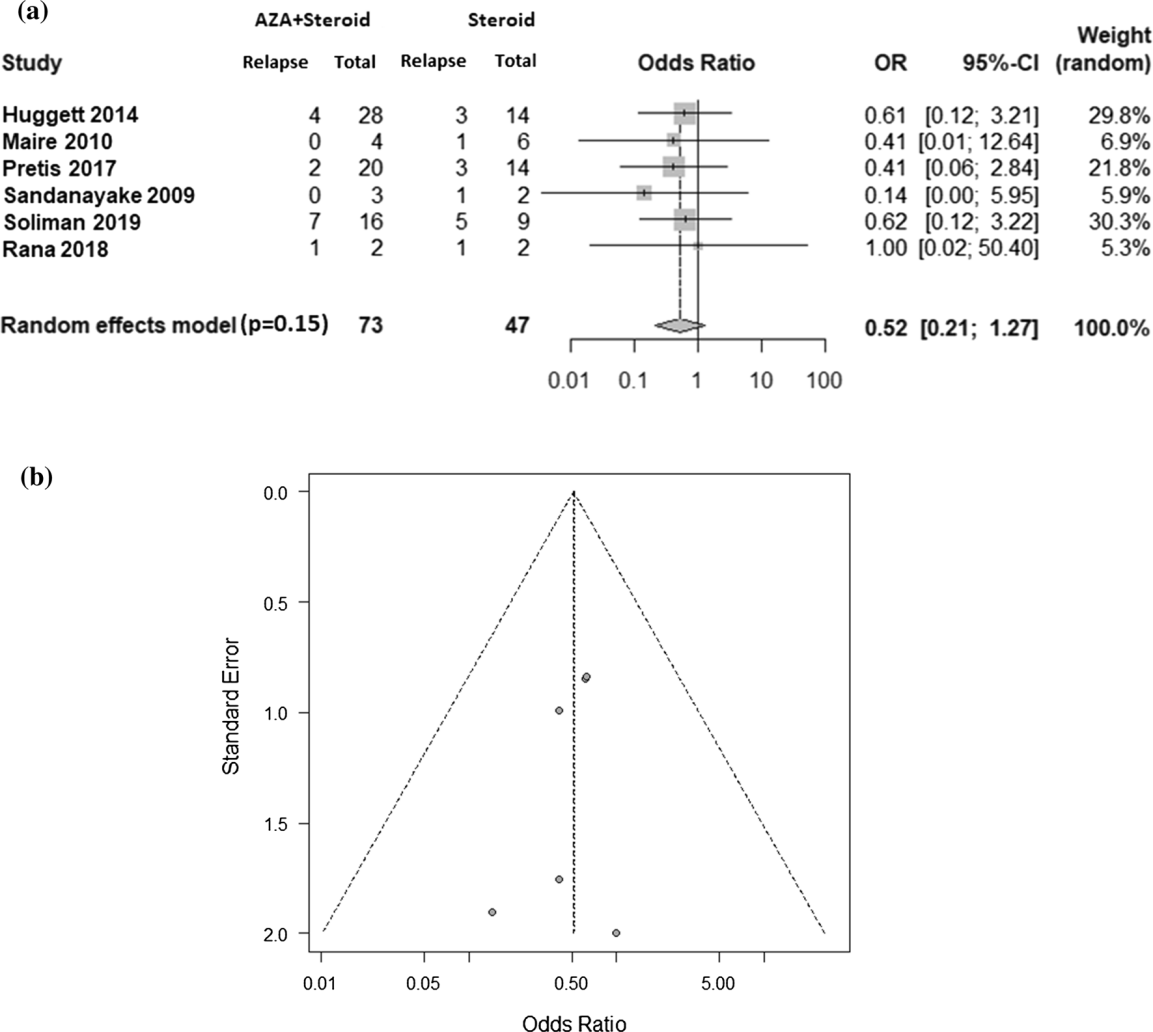
**A** Forrest plot showing the odds ratio of relapse in patients with AIP treated with AZA who experienced retreatment with steroids. The squares show the effect estimated from the single studies; the diamond shows the pooled result. **B** The funnel plot for relapse rate showed an approximately symmetrical appearance

Therefore, six studies were finally selected for meta-analysis. In these studies, 14/73 (19.2%) patients receiving AZA for refractory AIP relapsed. Meanwhile, 14/47 (29.8%) patients not receiving AZA exhibited relapse (Fig. 2A). The integrated odds ratio for rerelapse risk in patients receiving AZA was estimated as 0.52 (*p* = 0.15) using a random-effects model with the DerSimonian-Laird method. The results did not show statistical significance; however, the integrated odds ratio favored steroids with AZA compared to steroids without AZA. The results of the funnel publication bias plot for relapse rate exhibited an approximately symmetrical appearance, suggesting that the present analysis was absent of bias (Fig. 2B).

## Discussion

This systematic review and meta-analysis of collected data from 10 studies suggested the efficacy of AZA in preventing AIP relapse, which supports AZA as a maintenance treatment in patients with AIP who relapse upon the withdrawal of steroid therapy. The current data strongly suggest that AZA treatment is an alternative option in AIP patients who repeatedly relapse or are resistant to steroids.

Regarding remission induction for AIP, steroid treatment is the mainstay because several clinical studies have already reported high steroid responsiveness in AIP patients. A national multicenter survey in Japan [5] demonstrated a remission rate of 98% in AIP patients treated with 0.6 mg/kg of CS. An international multicenter study [32] reported that remission rates in AIP patients with type I and type II who received CS were 99.6% and 92.3%, respectively. Based on these cohort data, we initiated CS for patients with an initial diagnosis of AIP. The clinical challenge is that AIP patients typically relapse after steroid withdrawal or fail steroid weaning, despite the higher response rate to steroid treatment in AIP. In data pooled from three treatment studies in patients with AIP or IgG4-associated cholangitis (IAC), the overall relapse rate in AIP patients who received CS ranged from 27 to 53%. Japanese cohort data [5] demonstrated that the AIP patient relapse rate has been increasing each year according to Kaplan–Meier analysis. More than 30% of patients relapse, even when taking more than 5 mg of CS daily.

In this meta-analysis, we found that the initial dose of CS varied for each study, such as 30–40 mg/day, 0.6 mg/kg/day, and 1 mg/kg/day. Ikeura et al. reported that the initial dose of prednisone for AIP patients was 1 mg/kg of body weight per day for 2–3 weeks, and 17 (23%) out of 74 patients relapsed after withdrawal of CS treatment [22]. Compared to Kamisawa’s report [3], the relapse rate for first line CS treatment in this study was lower. They concluded that treatment with initial high-dose CS might reduce the risk of relapsed AIP. However, the risk of side effects related to CS increased as the cumulative total CS dose increased. This meta-analysis demonstrated the efficacy of AZA in addition to CS for preventing relapse and maintaining remission in AIP patients for whom the initial dose of CS was relatively low (i.e., 30 mg/day or 0.6 mg/kg/body). In this regard, AZA can contribute to reducing the total dose of CS in relapsed AIP patients.

In clinical practice, on-site physicians consider repeated steroid therapy to control most patients with AIP relapse because relapse typically responds to the reintroduction of an increased dose of CS. However, long-term use of low-dose CS and repeated induction with CS treatment contribute to an increased risk of side effects related to CS, such as vertebral fractures, osteonecrosis of the femoral head, and diabetes, because most AIP patients are elderly. Therefore, the establishment of a standard of care for managing relapse of AIP is clinically essential.

In addition to rheumatic disease, inflammatory bowel disease, and other diseases, there have been reports examining the efficacy of MMF, AZA, and 6MP in preventing AIP relapse [15, 38]. Interestingly, rituximab's therapeutic efficacy in AIP patients refractory to these immunomodulators has also been reported [38]. In this meta-analysis, we focused on the therapeutic preventive effect of AZA on AIP relapse because AZA is the most commonly used IM for AIP. Its utility has also been evaluated in other immune disorders. Maire et al. reported that four patients treated with AZA who were unresponsive to CS or who experienced CS weaning failure exhibited no relapse or adverse effects [14, Supplementary Fig. 3]. On the other hand, Xin et al. reported that eight of 34 patients who received reinitiation of steroids without AZA experienced repeated relapses [18, Supplementary Fig. 7]. They concluded that reinduction of CS might not be sufficient to prevent another relapse of AIP. On the other hand, there are several reports regarding AIP rerelapse despite additional AZA administration [13, 15, 17, 21, Supplementary Fig. 2,4,6,10].

However, in determining the efficacy of AZA treatment, we should consider the dose and how long AZA is appropriate for maintenance therapy of AIP. In fact, in this meta-analysis, we found that the dose of AZA varied for each study, such as 50 mg/day, 100 mg/day, and 2–2.5 mg/kg/day. All data used in the meta-analysis came from Western patients. Therefore, it is not appropriate to apply these doses to Asian patients with AIP. Based on the racial difference in nucleoside diphosphate-linked moiety X-type motif 15 (NUDT15) and thiopurine methyltransferase activity between Caucasians and Asian patients, monitoring their 6-thioguanine nucleotide (6TGN) levels is required to determine the optimal AZA dose for controlling AIP, which is also relevant in other autoimmune diseases. Unfortunately, none of the studies enrolled in the meta-analysis checked the 6-TGN levels to adjust the optimal AZA dose. Although this meta-analysis suggests the benefit of AZA in preventing AIP relapse compared to reinitiation of steroid therapy, determining the dose of AZA based on the 6-TGN level may further improve the efficacy of maintenance therapy for AIP patients.

Another issue is side effects related to AZA is that it causes significant side effects, such as pancreatitis, gastrointestinal symptoms (nausea and vomiting), liver injury, severe leukopenia, and hair loss. Among them, most physicians are concerned about pancreatitis. The risk of acute pancreatitis following AZA treatment is relatively low, despite the unknown frequency of AZA-induced pancreatitis in AIP patients. In clinical practice, given that an AIP patient develops pancreatitis during AZA treatment, it is difficult to determine whether AIP relapses or AZA has side effects. AZA-induced pancreatitis is an unpredictable and dose-independent adverse event affecting 2–7% of IBD patients treated with AZA [39]. There is a recent report indicating that the HLA-DQA1-HLA-DRB1 polymorphism is an important marker for AZA-induced pancreatitis risk [39]. Therefore, to avoid AZA-induced pancreatitis in Caucasians, an examination of HLA typing would be helpful. In 10 included studies for this meta-analysis, AZA-related side effects were observed in 21 (14.6%) of 144 patients. The side effects in AZA-treated patients were intolerance (*n* = 13), nausea (*n* = 4), dizziness (*n* = 1), hepatitis (*n* = 1), anaphylactic shock (*n* = 1), and myelosuppression (*n* = 1). However, there were no reports regarding severe leukopenia or hair loss related to AZA, which are observed in Asian patients. A strong association between NUDT15 gene polymorphism and thiopurines related to acute severe leukopenia or hair loss has been reported in people of Asian ancestry [40–42]. Patients who are homozygous and heterozygous for p. Arg139Cys (NUDT15 T/T and C/T genotype, respectively) have lower enzyme activity than those homozygous for the wild allele (C/C genotype), resulting in dose-dependent AEs [43]. We also know that the frequency of the high-risk genotype in Japanese people is approximately 1%. Therefore, initial screening for NUDT15 gene polymorphisms is useful for eliminating on-site physicians’ concerns about prescribing thiopurines. Therefore, genotyping for NUDT15 is essential in AIP patients of Asian descent for whom we consider AZA administration.

AIP can be subclassified into two subtypes, i.e., 1 and 2, according to unique pancreatic histopathologic patterns and its demographic profiles, clinical presentation, and natural history [44]. In this meta-analysis, we could not accurately classify AIP patients identified for this meta-analysis due to the few detailed descriptions regarding the clinical features and pathological findings of refractory cases in most of the included studies. Based on the rarity of relapse in type 2 AIP patients and the popular and long-standing association of the term “AIP” with what is now called “type 1 AIP”, we think that most of the patients included in this meta-analysis had type 1 AIP.

The results of this meta-analysis have several limitations. First, differences in patient background, study design, sample size, the severity of AIP, and treatment protocol, including the initial dose of CS for remission induction, might affect the quantitative analysis. We attempted to control for these differences and maintain study quality using scoring the quality assessment scale. Second, a relatively small number of studies included in this meta-analysis might have sampling bias. However, the funnel plot for relapse rate showed an approximately symmetrical appearance, suggesting that this meta-analysis was absent of any bias. Third, all studies included in this meta-analysis were observational studies. Differences in background factors between groups administered AZA and groups not administered AZA were not clearly described in each study; therefore, meta-regression analysis to examine the impact of moderator variables on study effect size could not be performed. Generally, it is appropriate to use survival analysis because relapse is an event observed during treatment; however, Cox regression analysis regarding treatment with azathioprine and relapse was not performed in referred papers for this meta-analysis. Instead, we applied the odds ratio, rather than the hazard ratio, as the effect measure in the meta-analysis. From this point of view, we cannot deny that the present analysis contains exploratory elements. In general practice, AZA was administered to steroid-dependent, steroid-refractory patients and those with AIP with multiple relapses [14, 19, 20]. Thus, the group with AZA included more patients with refractory AIP than that without AZA. Therefore, in this study, where adjustment for background factors was difficult, the outcome of interventional treatment with AZA could have been considered unfavorable. Nevertheless, it is be noted that this meta-analysis demonstrated that the integrated odds ratio for relapse risk in patients with AZA was estimated to be 0.52.

In conclusion, our data demonstrated the beneficial role of AZA in preventing relapse and maintaining remission of AIP. At present, there have been no RCTs and the use of AZA in patients with AIP is off-label worldwide. Therefore, RCTs including investigator-initiated clinical trials or advanced medical care are required to provide evidence for the efficacy of AZA in AIP.

## Supplementary Information

Below is the link to the electronic supplementary material.

Supplementary file1 (TIF 87 KB)

Supplementary file2 (TIF 169 KB)

Supplementary file3 (TIF 141 KB)

Supplementary file4 (TIF 88 KB)

Supplementary file5 (TIF 91 KB)

Supplementary file6 (TIF 78 KB)

Supplementary file7 (TIF 182 KB)

Supplementary file8 (TIF 114 KB)

Supplementary file9 (TIF 113 KB)

Supplementary file10 (TIF 90 KB)

Supplementary file11 (TIF 83 KB)

Supplementary file12 (TIF 80 KB)

Supplementary file13 (TIF 104 KB)

Supplementary file14 (DOCX 24 KB)
