# Supplementary figures and images for: The clinical efficacy of azathioprine as maintenance treatment for autoimmune pancreatitis: a systematic review and meta-analysis
Source: J Gastroenterol. 2021 Aug 24;56(10):869–80. doi: 10.1007/s00535-021-01817-9 (PMC8382580; doi:10.1007/s00535-021-01817-9)

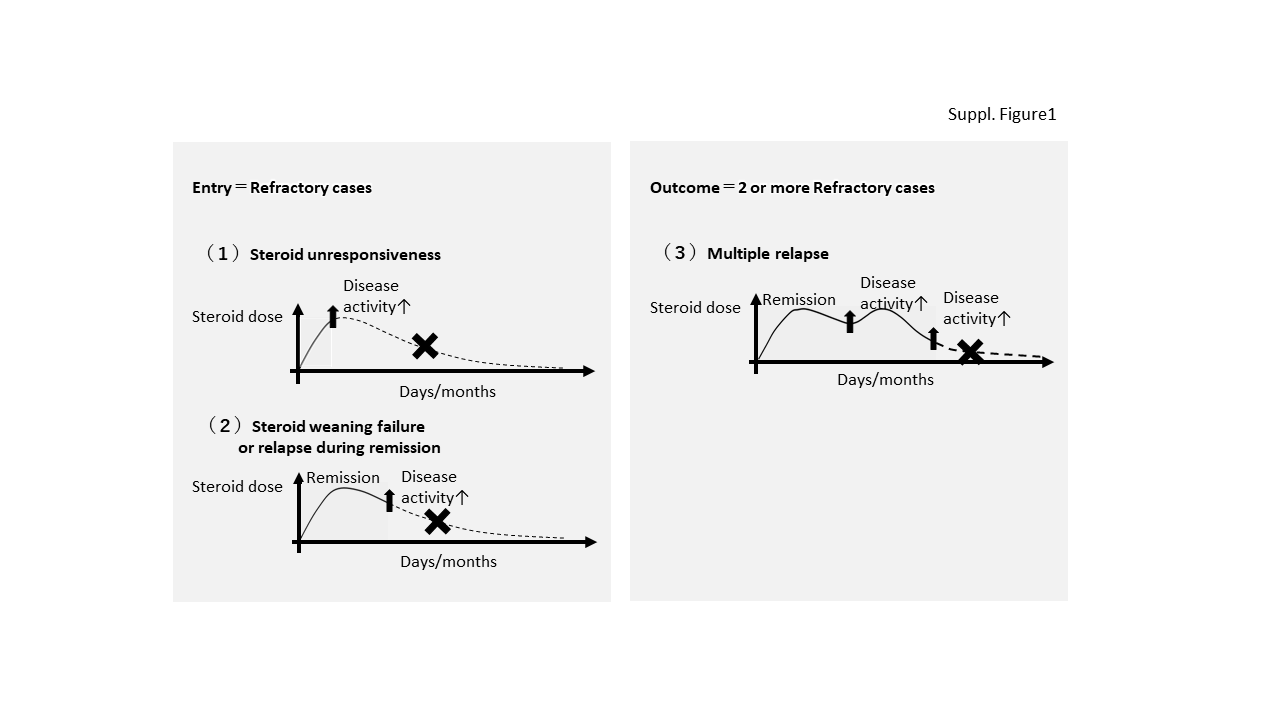

Supplement: Supplementary file 1 — Supplementary file1 (TIF 87 KB) [file 535_2021_1817_MOESM1_ESM.tif]

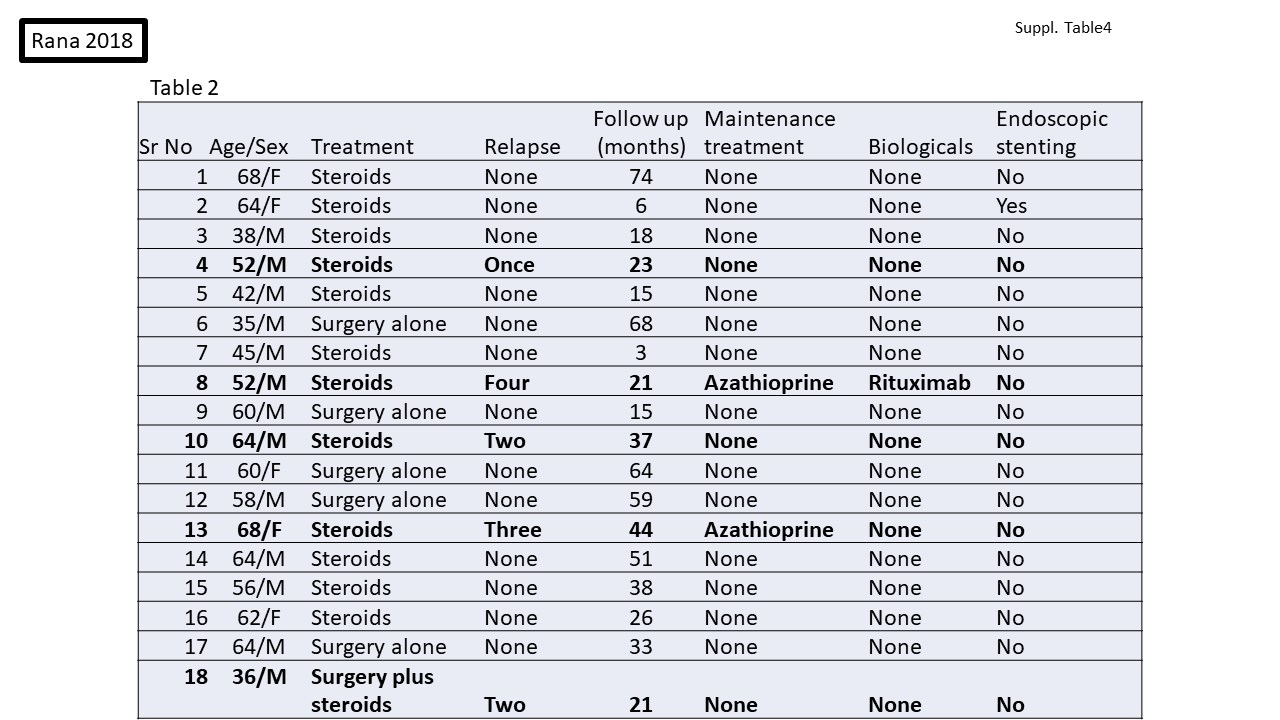

Supplement: Supplementary file 2 — Supplementary file2 (TIF 169 KB) [file 535_2021_1817_MOESM2_ESM.tif]

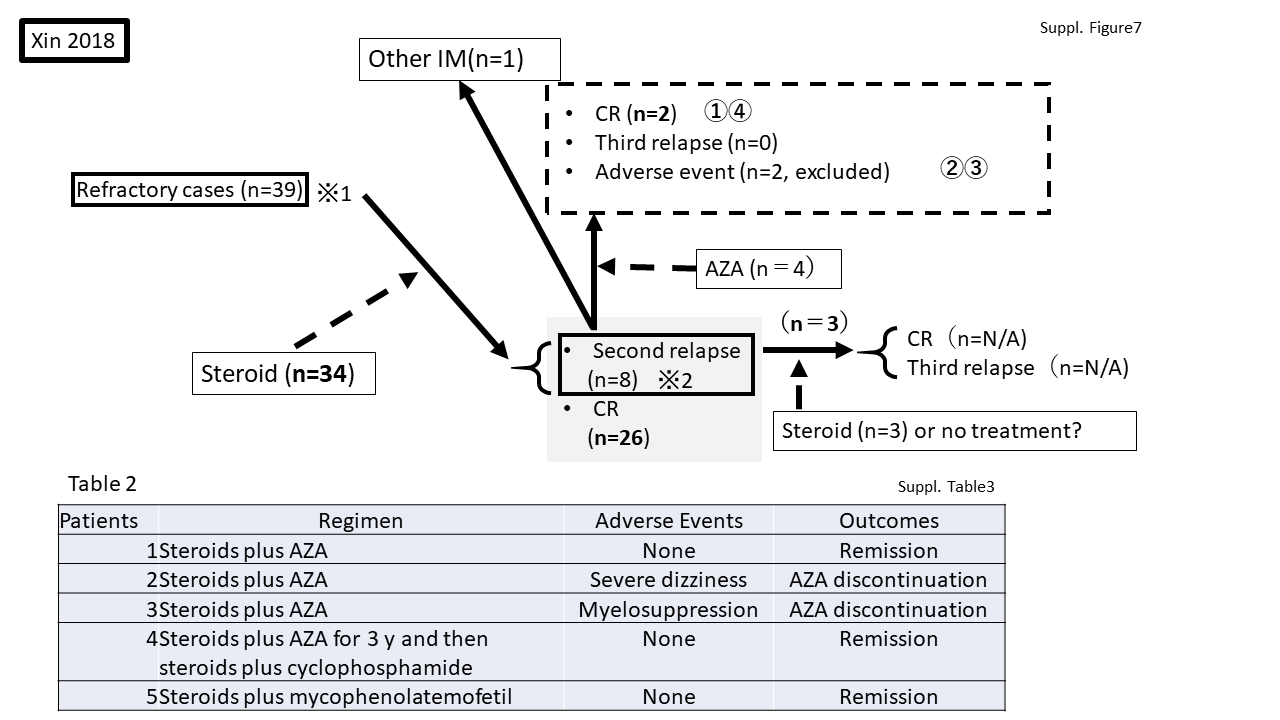

Supplement: Supplementary file 3 — Supplementary file3 (TIF 141 KB) [file 535_2021_1817_MOESM3_ESM.tif]

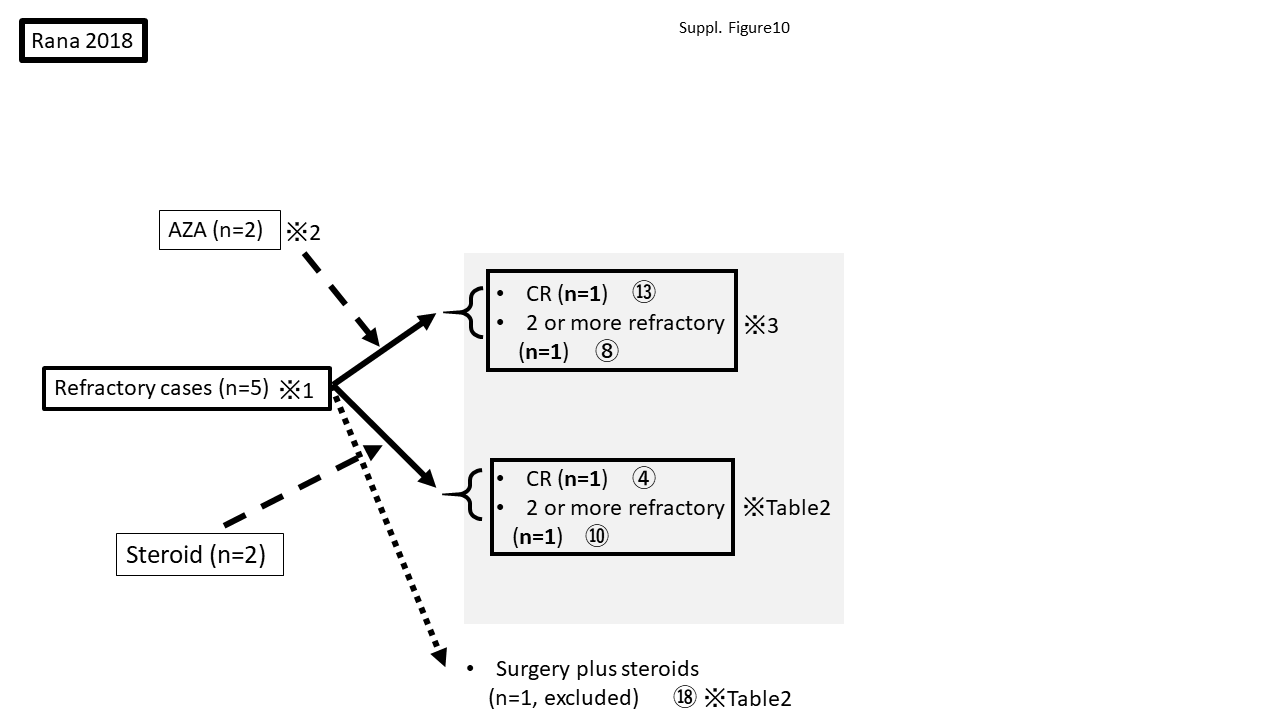

Supplement: Supplementary file 4 — Supplementary file4 (TIF 88 KB) [file 535_2021_1817_MOESM4_ESM.tif]

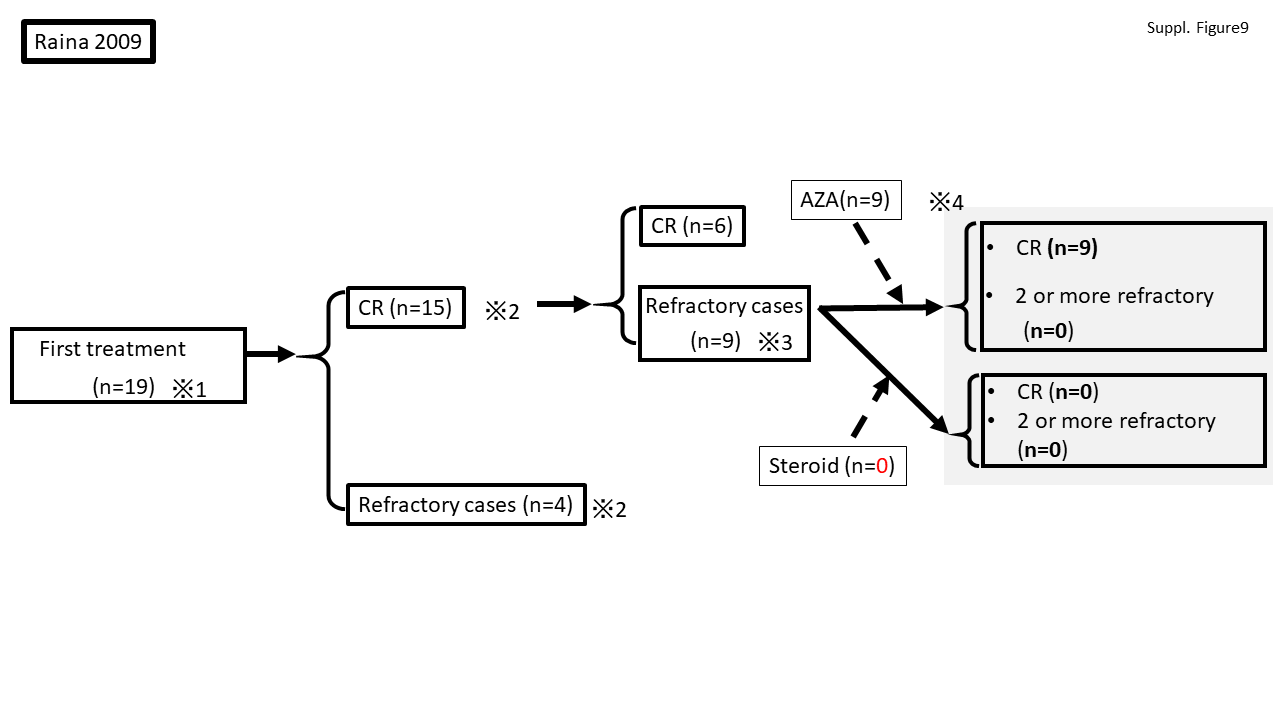

Supplement: Supplementary file 5 — Supplementary file5 (TIF 91 KB) [file 535_2021_1817_MOESM5_ESM.tif]

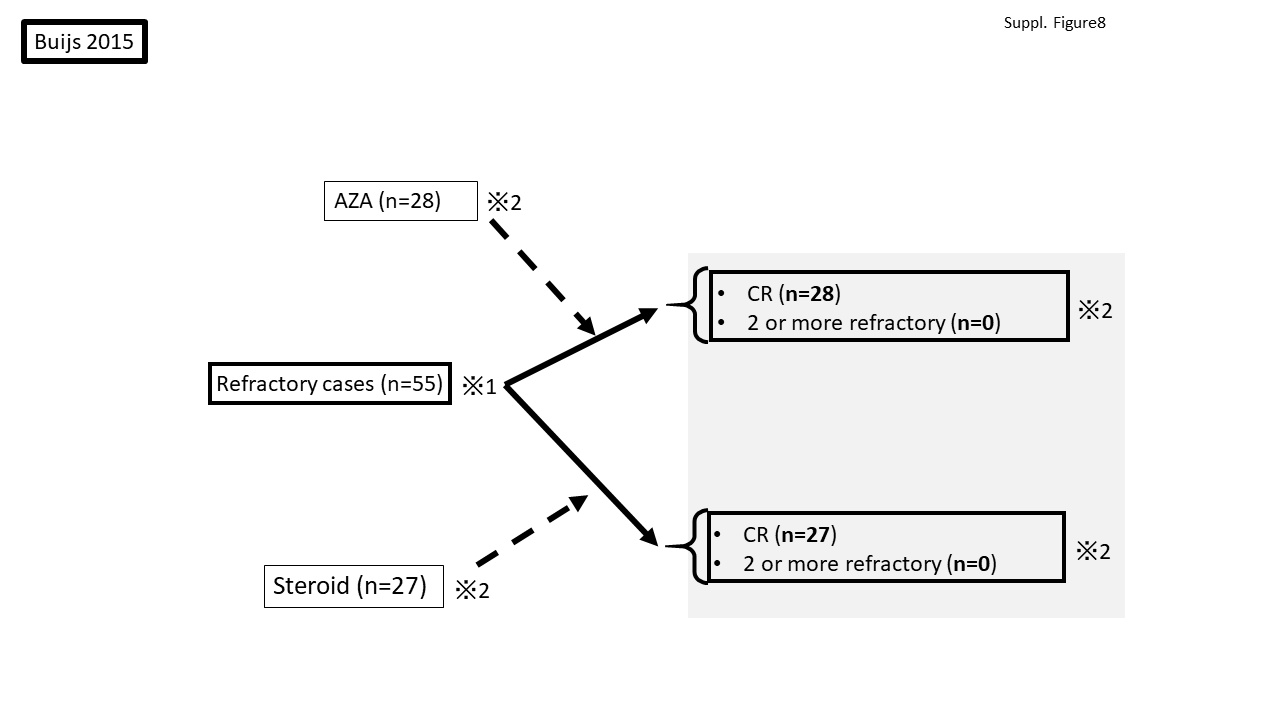

Supplement: Supplementary file 6 — Supplementary file6 (TIF 78 KB) [file 535_2021_1817_MOESM6_ESM.tif]

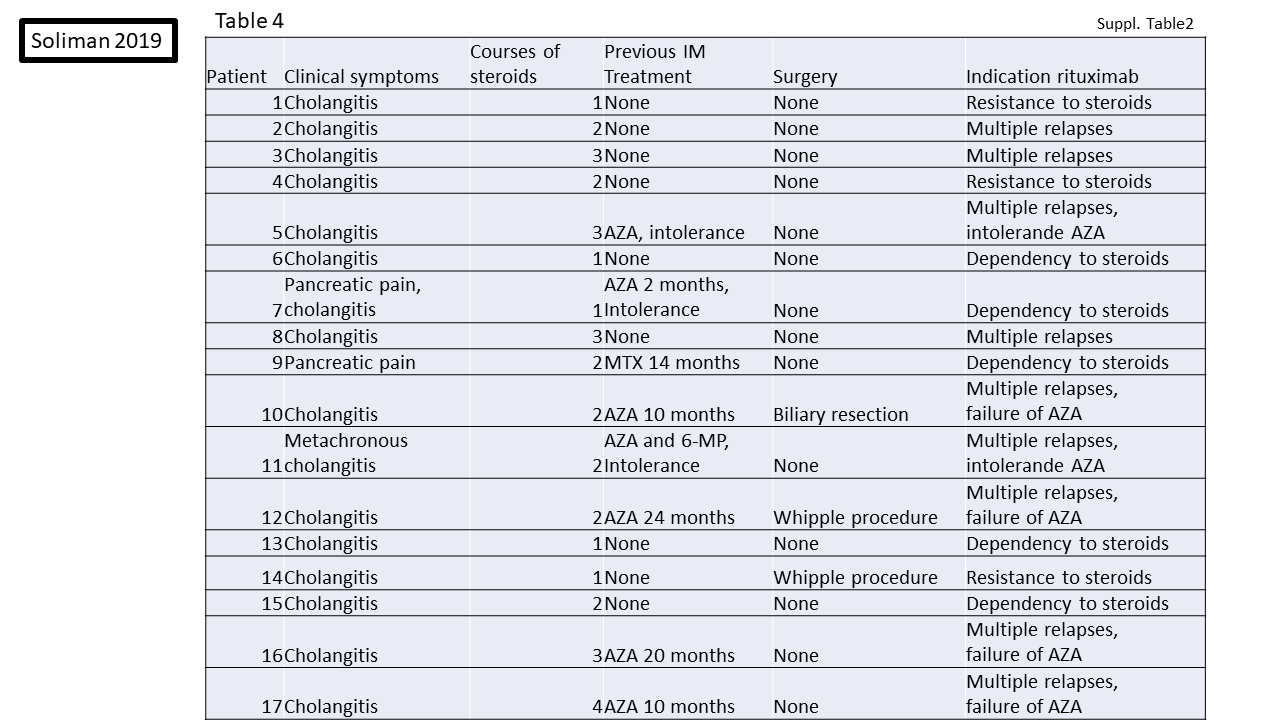

Supplement: Supplementary file 7 — Supplementary file7 (TIF 182 KB) [file 535_2021_1817_MOESM7_ESM.tif]

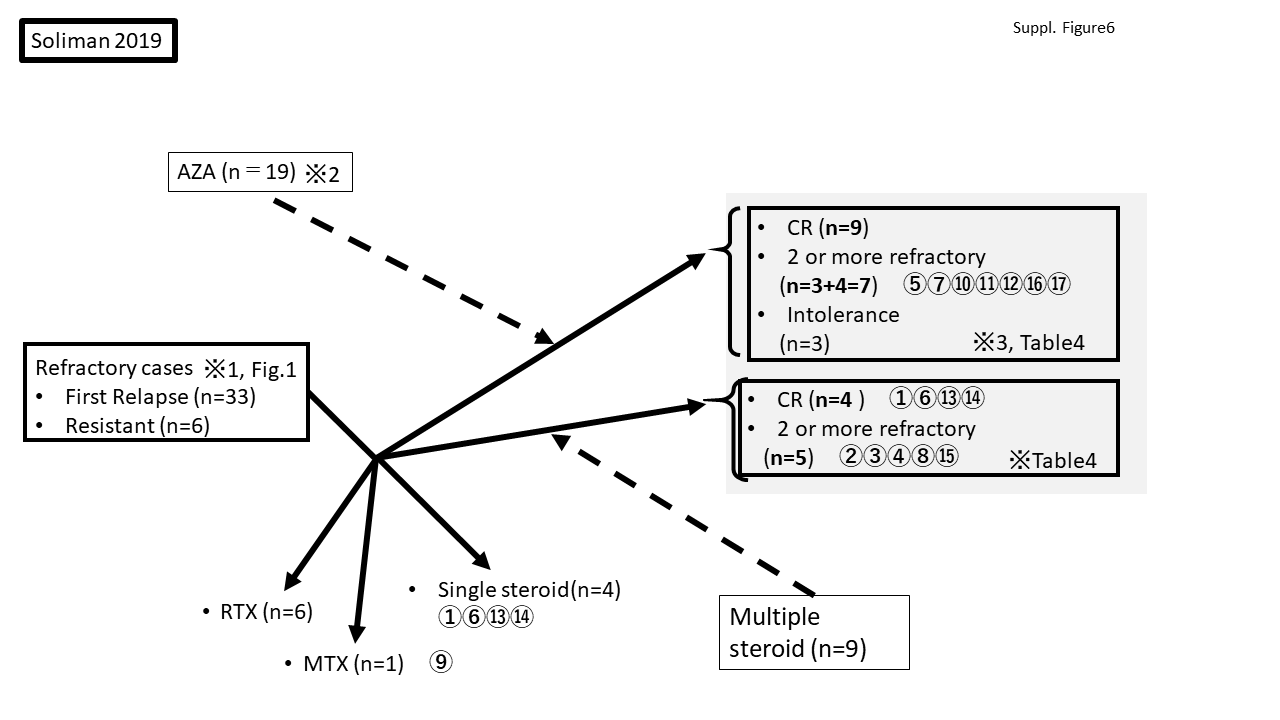

Supplement: Supplementary file 8 — Supplementary file8 (TIF 114 KB) [file 535_2021_1817_MOESM8_ESM.tif]

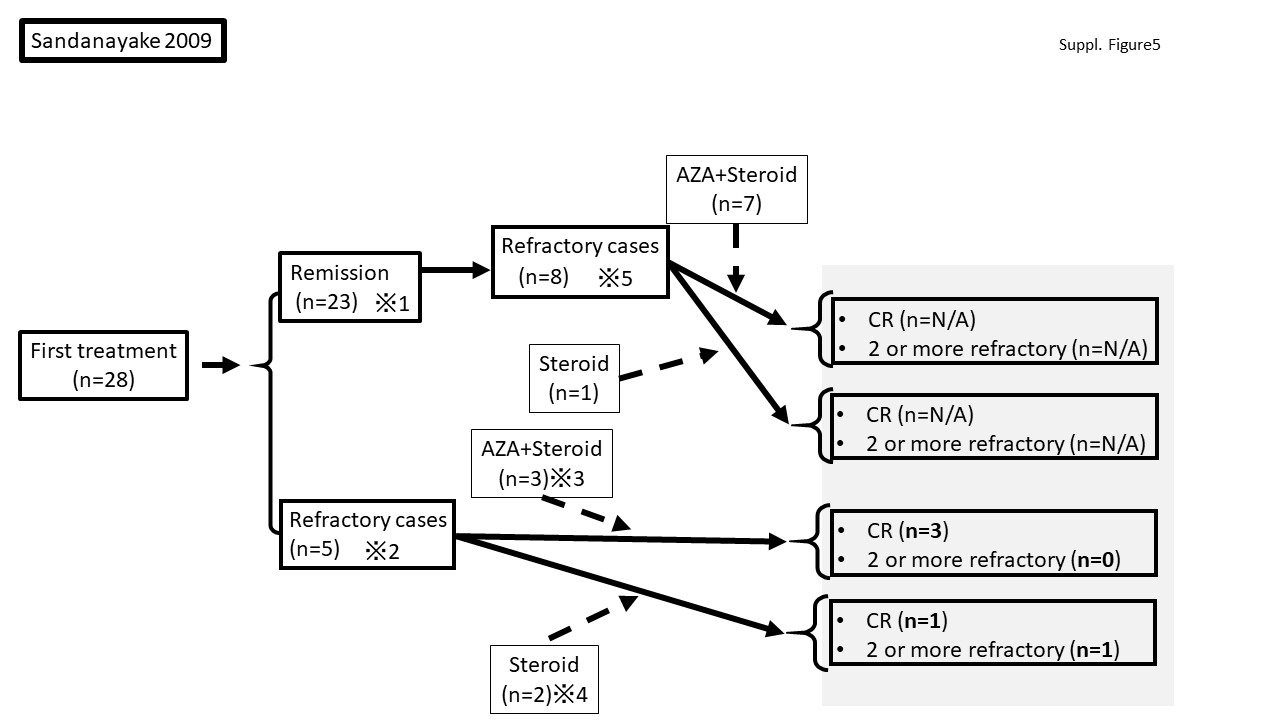

Supplement: Supplementary file 9 — Supplementary file9 (TIF 113 KB) [file 535_2021_1817_MOESM9_ESM.tif]

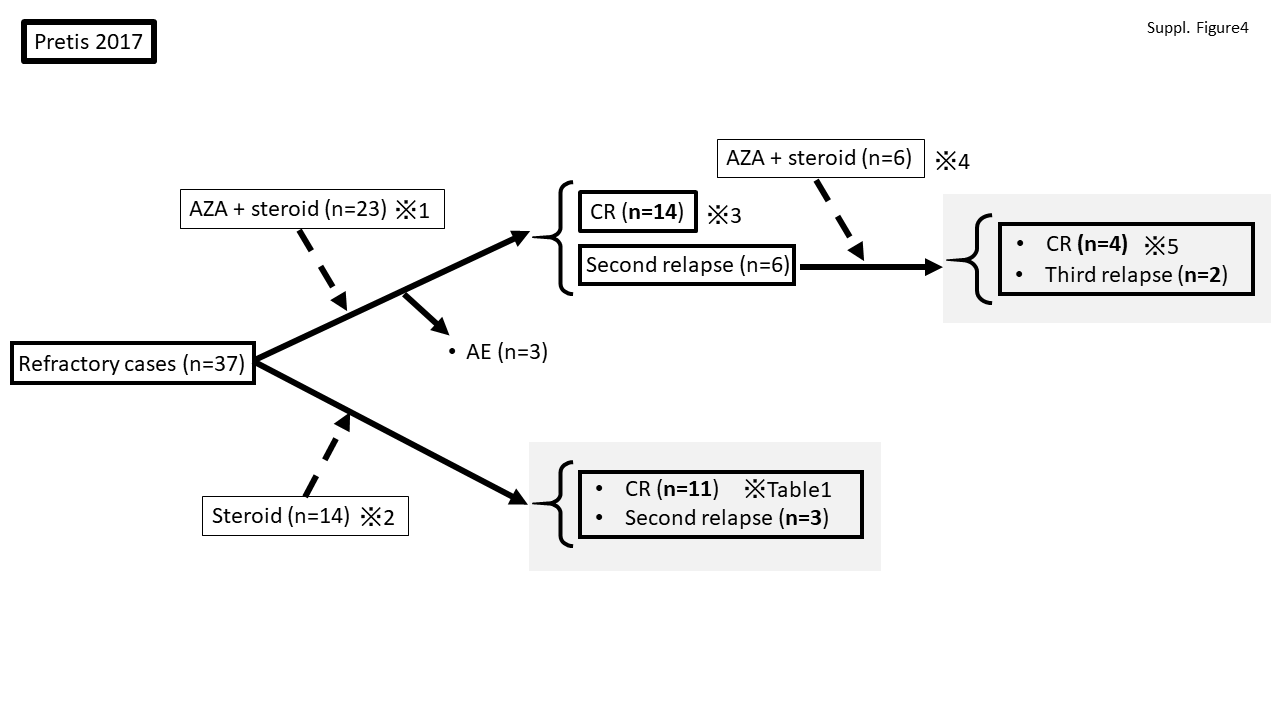

Supplement: Supplementary file 10 — Supplementary file10 (TIF 90 KB) [file 535_2021_1817_MOESM10_ESM.tif]

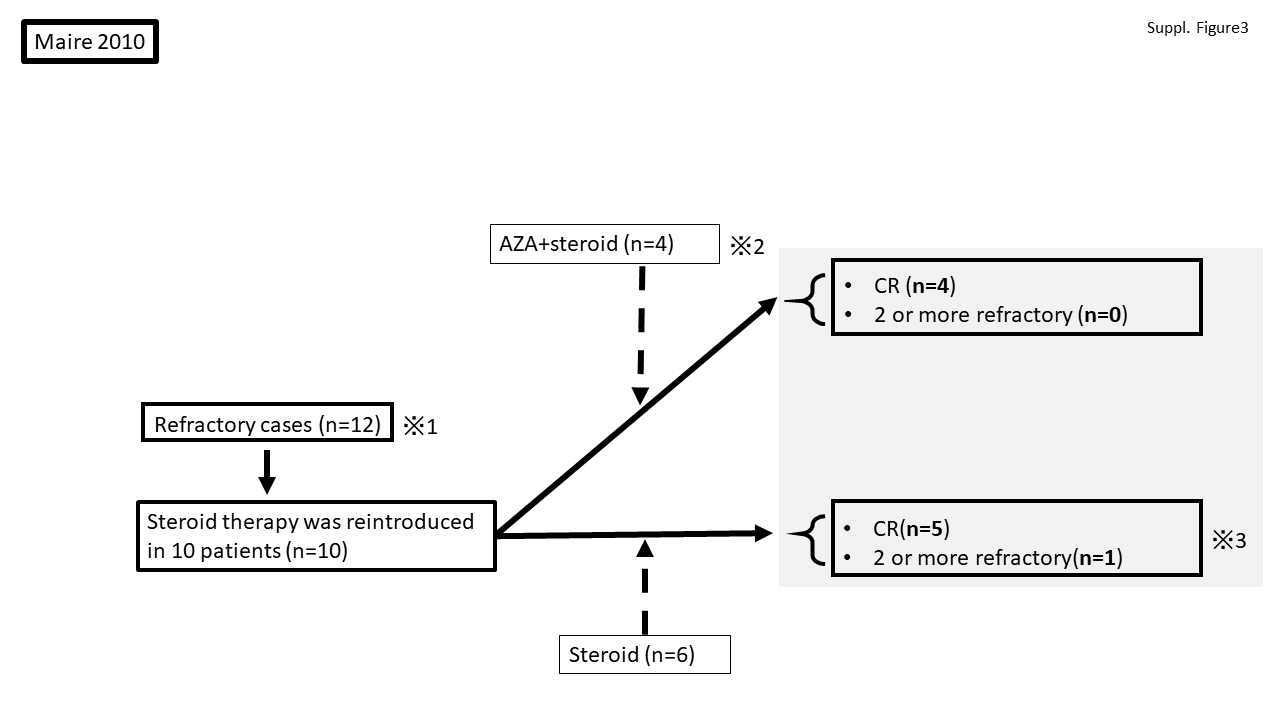

Supplement: Supplementary file 11 — Supplementary file11 (TIF 83 KB) [file 535_2021_1817_MOESM11_ESM.tif]

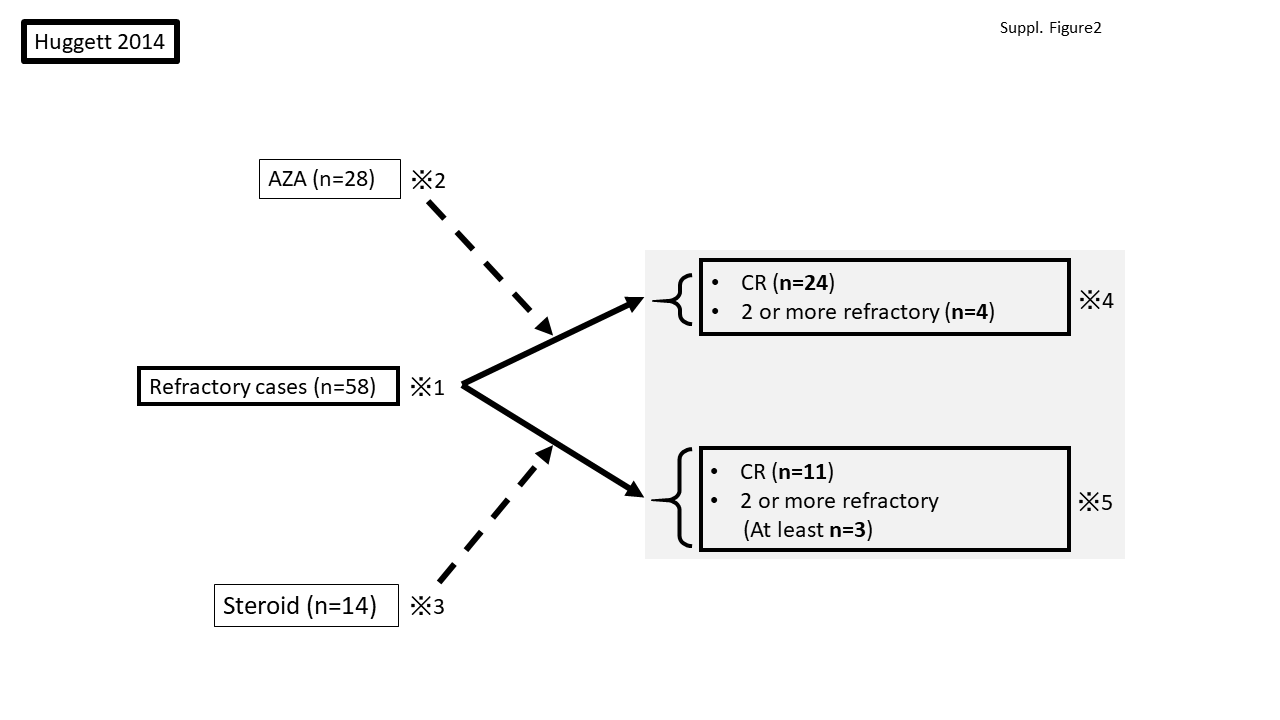

Supplement: Supplementary file 12 — Supplementary file12 (TIF 80 KB) [file 535_2021_1817_MOESM12_ESM.tif]

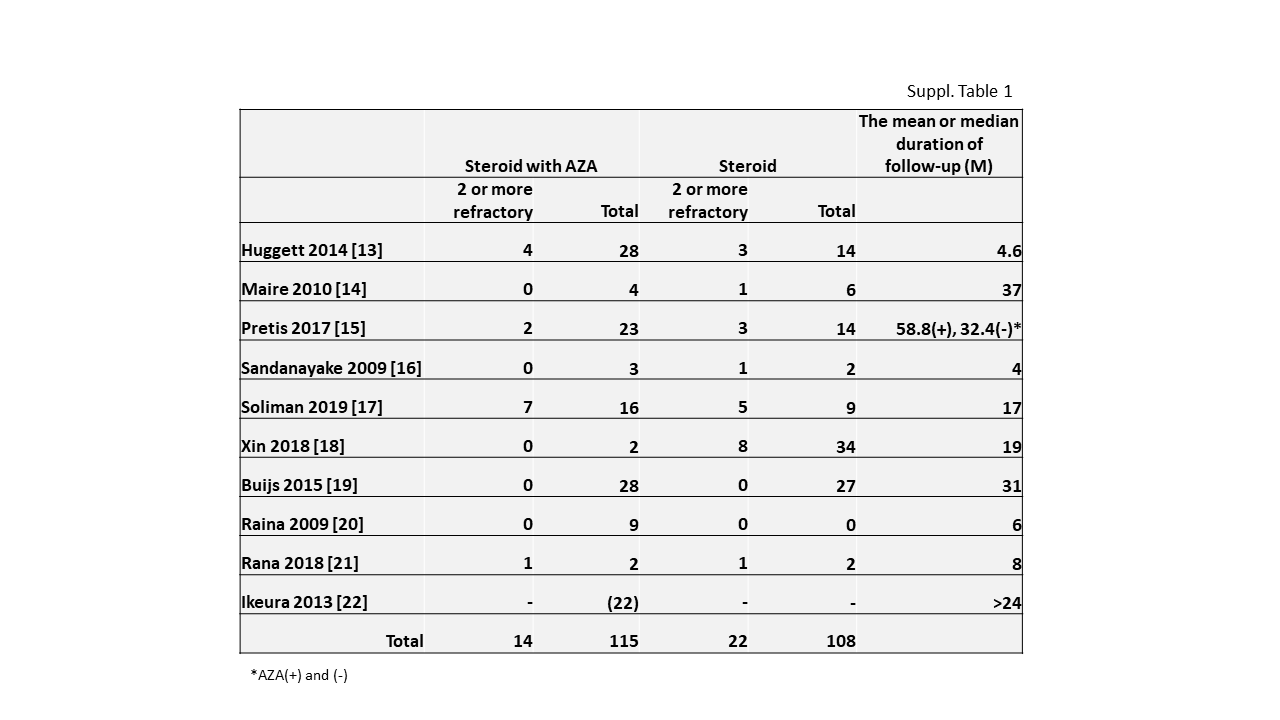

Supplement: Supplementary file 13 — Supplementary file13 (TIF 104 KB) [file 535_2021_1817_MOESM13_ESM.tif]
