# Supplementary material for: The clinical efficacy of azathioprine as maintenance treatment for autoimmune pancreatitis: a systematic review and meta-analysis
Source: J Gastroenterol. 2021 Aug 24;56(10):869–80. doi: 10.1007/s00535-021-01817-9 (PMC8382580; doi:10.1007/s00535-021-01817-9)
Supplement: Supplementary file 14 — Supplementary file14 (DOCX 24 KB) [file 535_2021_1817_MOESM14_ESM.docx]

**Supplement**

**Suppl. Fig. 1:** Definition of refractory patients and 2 or more refractory patients in this study. Refractory means (1) steroid unresponsiveness or (2) steroid weaning failure or relapse during remission; on the other hand, 2 or more refractory means (3) multiple relapses.

The details of the patient numbers used in this analysis are shown in Supplementary Figures 2-10 and Supplementary Tables 1-4.

**Suppl. Fig. 2:** Huggett et al. [13] reported that fifty-eight patients (50%) relapsed post steroid treatment at a median time of 4.6 (0 – 64) months after stopping the first course of steroids. Seventy-six percent of patients who relapsed received additional second-line immunosuppression, with the remainder being treated solely with a second course of oral corticosteroids. The usual approach from both centers was to treat with a further course of steroids and add azathioprine (to a dose of 2 mg/kg/day), which was started in 41 patients. Except for 13 patients who were intolerant of AZA, 28 patients received AZA + CS therapy. Of these, 8 rerelapsed; however, in those eight patients who relapsed on azathioprine, corticosteroids were restarted in all with an increased dose of azathioprine (n = 4) or a change in immunosuppression (n = 4). Twenty-four patients remained on immunomodulatory monotherapy at the end of the study without the requirement for additional corticosteroids. Therefore, we thought four patients who received an increased dose of AZA experienced long-term remission without re-relapsing. Finally, we judged that 4/28 patients who received AZA + CS therapy rerelapsed.

On the other hand, fourteen relapsed patients (24%) did not use immunomodulators, that is, these patients received CS therapy alone. In addition, two patients who were intolerant of azathioprine were treated with long-term corticosteroids. Therefore, we thought sixteen relapsed patients received reinitiation of steroid therapy. Four patients died due to IgG4-RD (pulmonary fibrosis; 2, end-stage liver disease; 1, and autoimmune encephalitis; 1). Of these 4 patients, only one patient received treatment with steroids and immunosuppression; that is, the patient was judged as having one rerelapsed after repeat steroid therapy (one patient developed rapidly progressive biliary structuring disease with progressive jaundice and died from cholangitis before liver transplantation). One patient with biopsy-proven multiorgan IgG4-RD developed a rapidly progressive encephalitic illness, and a diagnosis of autoimmune encephalitis was made on the basis of characteristic features of encephalitis on imaging (gadolinium magnetic resonance imaging) and cerebrospinal fluid analysis. This patient died despite treatment with high-dose steroids and immunosuppression. Two patients died of pulmonary fibrosis, one died of pulmonary embolism, and one died of pneumonia.

※1 (p1678, Right, Line3): Fifty-eight patients (50 %) relapsed post steroids, at a median time of 4.6 (0 - 64) months after stopping the first course of steroids, with two of these patients relapsing while taking steroids.

※2 (p1678, Right, Line16): The usual approach from both centers was to treat with a further course of steroids and add in azathioprine (to a dose of 2 mg/kg/day), which was started in 41 patients. Of these (n = 41), 8 relapsed and 13 were intolerant of Azathioprine.

※3 (p1678, Right, Line14): Seventy-six percent of patients who relapsed received additional second-line immunosuppression, with the remainder being treated solely with a second course of oral corticosteroids (58 X (1-0.76) = 14).

※4 (p1678, Right, Line26): In those eight patients who relapsed on azathioprine, corticosteroids were restarted in all with an increased dose in azathioprine (n = 4), or change in immunosuppression (n = 4). Twenty-four patients remained on immunomodulatory monotherapy at the end of the study, without requirement for additional corticosteroids.

※5 (p1680, Left, Line3): One patient developed rapidly progressive biliary structuring disease with progressive jaundice, and died from cholangitis before liver transplantation.

(p1680, Right, Line5): Two patients died of pulmonary fibrosis.

**Suppl. Fig. 3:** Maire et al. [14] reported that twelve patients (4/12 in the surgical group and 8/26 in the steroid group) (27%) experienced relapse, presenting as obstructive jaundice (n = 4), acute pancreatitis (n = 2), pancreatic pain (n = 5), or extrapancreatic autoimmune manifestation (n = 1). Two patients presented with pancreatic pain rapidly resolving with analgesics, suggesting that these patients also relapsed to AIP. Of these 14 patients, steroid therapy was reintroduced in 10 patients and was effective in 9 of them (90%). Azathioprine was started in four patients due to steroid unresponsiveness (n = 1) or steroid weaning failure (n = 3). The median duration of treatment with azathioprine was 37 (range, 20 – 47) months, suggesting that these four patients received long-term maintenance without relapsing.

※1 (p153, right, line18): Twelve patients experienced a relapse, presenting as obstructive jaundice (n = 4), acute pancreatitis (n = 2), pancreatic pain (n = 5), or extrapancreatic autoimmune manifestation (n = 1).

※2 (p154, left, line3): Azathioprine was started in four patients, because of steroid unresponsiveness (n = 1) or steroid weaning failure (n = 3).

※3 (p154, left, line2): Steroid therapy was reintroduced in 10 patients, effective in 9 of them (90%).

**Suppl. Fig. 4:** Pretis et al. [15] reported 37 patients experienced first relapse (Table 1). AZA was administered in 23 out of 37 patients who experienced first relapse. Three patients required AZA discontinuation within two months after starting because of adverse events: hepatitis, anaphylactic shock, nausea and body weight loss. Twenty patients were therefore evaluated for outcome. Fourteen out of 20 patients (70%) reached and maintained complete disease remission during follow-up, while 6 out of 20 (30%) relapsed after 24±15.4 months. Patients who presented recurrences were all retreated with another course of steroids followed by complete tapering and continued AZA therapy. Two out of six patients (33%) had a second relapse after respectively 11 months and 22 months and are in screening for RTX treatment. The other four patients did not have a second relapse after a mean follow-up time of 19±10 months. On the other hand, according to Table 1, re-steroid without AZA was administered in 14 out of 37 patients who experienced first relapse. Among these, 11 patients did not have a second relapse.

※1 (p2, Right, Line32) : Twenty-three patients in AZA^+^ group and 97 in AZA^-^ group were compared.

(p2, Right, Line45) : The indications for the maintenance therapy with AZA were relapse of AIP in 18 patients (78%) , extra pancreatic involvement in 3 (13%) and markedly increase of serum IgG4 after steroid treatment in 2 (9%).

※2 (p3, Right, Line35) : Among the 97 AZA- patients, 14 experienced a relapse of the disease.

※3 (p3, Left, Line5) : Fourteen out of 20 patients (70%) reached and maintained complete disease remission during follow-up, while 6 out of 20 (30%) relapsed after 24±15.4 months.

※4 (p3, right, Line9) : Patients who presented recurrences were all retreated with another course of steroids followed by complete tapering and continued AZA therapy.

※5 (p3, right, Line11) : Two out of six patients (33%) had a second relapse after respectively 11 months and 22 months and are in screening for RTX treatment. The other four patients did not have a second relapse after a mean follow-up time of 19±10 months.

**Suppl. Fig. 5:** Sandanayake et al. [16] reported that all 28 patients exhibited a disease response within 4 – 6 weeks of starting steroids. Of the 23 patients who achieved remission, 8 (35%) relapsed at a median of 4 months (range, 1 - 29 months) after ceasing the initial course of prednisolone. All 8 patients who relapsed commenced on 30 mg prednisolone at the time of their relapse and commenced on AZA. Six of the relapsed patients (75%) were able to cease their second course of steroids at a median of 6 months (range, 3 – 9 months). Patients 10 and 22 relapsed recently and began a second course of prednisolone and commenced AZA. Therefore, we regarded these two patients as rerelapse patients with AZA + CS. On the other hand, none of the relapsed patients were treated with the reinitiation of steroids without AZA.

※1 (p1092, Left, Line12): Of the 23 patients who achieved remission, 8 (35%) relapsed at a median of 4 months (range, 1–29 months) after ceasing the initial course of prednisolone.

※2 (p1092, Left, Line28): In 5 patients it was not possible to wean and stop the initial predonisolone course as a result of a flare of their disease.

※3: Three patients were commenced on AZA, with a target dose of 2mg/kg. Patient 21 and 2 were subsequently weaned off steroids after 4 and 14 months and remain in remission 11 and 22 months, respectively, on AZA monotherapy (Table3). Patient 3 was treated with AZA and 20mg predonisolone. His deranged liver biochemistry, biliary strictures, and associated elevated creatinine responded and did not flare on weaning steroids.

※4 (p1093, Left, Line16): Patient 14 developed neurologic deterioration with global cerebral dysfunction, while receiving predonisolone 5mg. Imaging suggested an immune-mediated encephalopathy. No significant response was seen to high-dose immunosuppression, including steroids, and the patient subsequently died.

※5 (p1092, Right, Line15): All 8 patients who relapsed were commenced on 30 mg prednisolone at the time of their relapse (Table 2). Blood thiopurine S-methyltransferase levels were measured (normal levels in all), and 7 of 8 patients were also commenced on 1 mg/kg azathioprine (AZA) daily, with a target dose of 2 mg/kg.

**Suppl. Fig. 6:** Soliman et al. [17] reported that 21 patients were treated with IMs: AZA (n = 19) and methotrexate (n = 2). According to Figure 1, 18 relapsed patients were treated with AZA. The treatment was effective in preventing relapse for 14 patients. On the other hand, of 33 type 1 AIP patients who experienced multiple relapses or steroid dependencies, 9 patients received multiple courses of CS without the usage of IMs, suggesting that all 9 patients achieved long-term remission. Of the 33 patients mentioned above, 6 patients received rituximab therapy. According to Table 4, of the 17 patients with type 1 AIP treated with rituximab, five patients (No. 2, 3, 4, 8, 15) received multiple courses of CS therapy without the usage of IMs. Therefore, we judged that these five patients relapsed with reinitiation of steroid therapy.

※1 (p1076, Left, Line12): After this first line of treatment, relapse occurred in 33 patients with type 1 AIP (46.5%) versus four patients with type 2 AIP (12.5%; p < 0.001).

※2 (p1076, Right, Line5): In total, 21 patients were treated with IMs: azathioprine (n = 19), and methotrexate (n = 2). Indications were a relapsing disease for 18 patients and resistance to steroids for three.

※3 (p1076, Right, Line13): Four patients had relapse with cholangitis (cholestasis or jaundice) and were treated with rituximab, and three stopped azathioprine because of intolerance (nausea and vomiting); two of these patients were switched to rituximab. At the end of the maintenance treatment with azathioprine after 3 years, 3/12 (25%) patients had a relapse of the disease, within 6 months for two of them.

**Suppl. Fig. 7:** Xin et al [18] reported on 39 patients with relapse, and 1 and 3 patients received surgery and conservative treatment, respectively. For the other 34 (87.2%) patients, repeated steroid treatment was given with the same initial dose. In follow-up, 8 patients experienced 2 to 5 relapses. AZA was administered in 4 out of 8 patients who experienced multiple relapses. With the exception of 2 patients who required discontinuing AZA due to adverse events, 2 patients successfully achieved more than 1-year remission.

※1 (p1111, Right, Line34): In 39 patients with relapse, 1 and 3 patients received surgery and conservative treatment, respectively. Moreover, a 79-year-old patient died due to advanced bladder carcinoma during follow-up. For the other 34 patients, repeated steroid treatment was given with the same initial dose but longer induction and tapering duration.

※2 (p1112, Left, Line3): In follow-up, 8 patients experienced 2 to 5 relapses, among which 5 patients received steroids plus immunomodulator (IM) (Table 2).

**Suppl. Fig. 8:** Buijs et al. [19] reported that fifty-five patients experienced relapse, all relapses were successfully treated with a restart of steroid therapy, and in 28 patients, azathioprine was added. None of these 28 patients rerelapsed. On the other hand, according to the treatment protocol, 27 relapsed patients for whom AZA was not added were treated with CS. However, the number of rerelapsed patients was unclear.

※1 (p1067, Right, Line1): Fifty-five patients experienced a relapse.

※2 (p1067, Right, Line10): All relapses were successfully treated with a restart of steroid therapy, and in 28 patients, azathioprine was added.

**Suppl. Fig. 9:** Raina et al. [20] reported that nineteen patients received corticosteroids as the initial treatment. Within 12 weeks of initiating the therapy, a complete response was observed in 15 (79%) patients, and an incomplete response was observed in 4 (21%) patients. Among the 15 patients with a complete response, 9 experienced recurrence within 8 - 12 weeks of steroid withdrawal. Recurrences were treated with corticosteroids during the acute flare-up, and azathioprine was added for long-term immunosuppression. All nine patients responded and were maintained on long-term azathioprine. Three patients out of 9 experienced more than one relapse; however, disease flares were controllable by adjusting the dose of corticosteroids without alternative drug addition. Therefore, we thought all relapsed patients were successfully treated with AZA + CS. On the other hand, steroid treatment without AZA was reinitiated in no relapsed patients.

※1 (p7, Line15): The remaining 19 patients received corticosteroids as the initial treatment.

※2 (p7, Line17): A complete response was observed in 15 and incomplete response in 4 patients.

※3 (p7, Line18): Among the 15 patients with a complete response, 9 had a recurrence within 8–12 weeks of steroid withdrawal.

※4 (p7, Line21): Recurrences were treated with corticosteroids during the acute flare-up and azathioprine was added for longterm immunosuppression. All nine patients responded and are being maintained on long-term azathioprine.

**Suppl. Fig. 10:** Rana et al. [21] reported that the disease relapsed in 5/13 (38%) patients who responded to initial steroids. Three or more relapses were observed in only 2/13 (15%) patients, both of whom were put on maintenance therapy with azathioprine. One of these patients relapsed for a fourth time, despite being on azathioprine. On the other hand, according to Table 2, relapse after initial CS treatment occurred in 5 patients (No. 4, 8, 10, 13, and 18). Of these 5 patients, AZA was not administered in three patients (No. 4, 10, and 18). One patient experienced single relapse, and two patients experienced multiple relapses; that is, two out of three patients experienced rerelapse with reintroduction of steroid therapy.

※1 (p509, Right, Line19): The disease relapsed in 5/13 (38%) patients who responded to initial steroids over a mean follow-up period of 34.2±21.6 weeks.

※2 (p509, Right, Line22): Three or more relapses were observed in only 2/13 (15%) patients, both of whom were put on maintenance therapy with azathioprine.

※3 (p509, Right, Line24): One of these patients relapsed for a fourth time, despite being on azathioprine, and showed an inadequate response to steroids.
